# Society Meetings

**Published:** 1899-06

**Authors:** 


### SOCIETY MEETINGS.

The Third District Branch of the New York State Medical Association, will be held at Elmira, Thursday, June 1, 1899, under the presidency of Dr. F. D. Reese. An interesting program has been prepared and a good attendance is expected. Dr. L. W. Higgins, of Cortland, is the secretary.

---

The Ohio State Medical Society held its 54th annual meeting at Springfield, May 10, 11 and 12, 1899, under the presidency of Dr. N. R. Coleman, of Columbus. A large and interesting program was disposed of and the meeting was one of the best in the history of the society.

THE Buffalo Academy of Medicine held meetings during the month of May, 1899, as follows:

*Section on Surgery.*—Tuesday evening, May 2d, program: Impressions of a medical man in Cuba, Marshall Clinton, 1st Lieut. and Assistant Surgeon 202 Infantry, U. S. V.

*Section on Medicine.*—Tuesday evening, May 9th, program: The use of alcohol in the treatment of disease, Joseph W. Grosvenor; discussion by Henry R. Hopkins, A. L. Benedict, E. L. Frost, Sydney A. Dunham, J. Grafton Jones.

*Section on Pathology.*—Tuesday evening, May 16th, program: Aortic anuerism, with specimens, also specimens of gall stones, Joseph P. F. Burke; exhibit of gall stones, by Dr. Carr.

*Section on Obstetrics.*—Tuesday evening, May 23d, program: Painful menstruation, C. E. Congdon. Physicians, surgeons, and fees, S. Y. Howell.

---

THE Medical Society of the County of Chemung held its regular meeting at Elmira, May 16, 1899. The following was the program: J. W. Gee, president of the society read the annual address; C. G. R. Jennings, Report of an interesting case; F. W. Ross, Typhoid fever among raw troops; Wm. C. Krauss, of Buffalo, N. Y., read a paper on nasal and cranial diseases, their close relation.

---

THE ninth annual meeting of the National Association of Homeopathic Medical Examiners, will be held at Atlantic City, New Jersey, on Monday afternoon, June 19, 1899.

Members and ex-members of state medical examining boards, of either school of medicine, are cordially invited to attend this meeting of the association. H. M. Paine, secretary.

---

THE Medical Society of the County of Erie will hold its regular semi-annual meeting at the Buffalo Academy of Medicine parlors in the Market Arcade, Tuesday, June 13, 1899, at 10 o'clock a. m., under the presidency of Dr. J. B. Coakley, of Buffalo. An interesting program is in preparation.

---

THE American Medico-Psychological Association held its fifty-fifth annual meeting at the Waldorf-Astoria, New York, May 23, 24, 25 and 26, 1899.

THE American Dermatological Association held its twenty-third annual meeting at Hotel Walton, Philadelphia, May 30 and 31 and June 1, 1899.

---

THE Esculapian Club held its regular meeting, Thursday, May 18, 1899, at the residence of Dr. W. B. Salesman, 332 E. Ferry street, corner Northland avenue. Dr. A. J. Colton read a paper entitled, Infantile eclampsia.

---

THE ninth annual meeting of the American Electro-therapeutic Association will be held in Washington, D. C., on September 19, 20 and 21, 1899, under the presidency of Dr. F. B. Bishop, of Washington.

Quite a number of papers of more than ordinary scientific value have been promised and the committee of arrangements ensures the members a very entertaining and pleasurable meeting. Aside from the sessions of the Association, the committee has completed arrangements for a trip to Mount Vernon, one to Arlington, and several other social features. The headquarters of the association will be at Willard's Hotel, where special rates will be given to members and their families during the meeting.

---

THE American Academy of Medicine will hold its twenty-fourth annual meeting at Columbus, O., June 3 and 5, 1899, in the Assembly Hall of the Hotel Chittenden, under the presidency of Dr. Edward Jackson, of Denver, Colorado. The secretary, Dr. Charles McIntire, of Easton, Pa., has prepared a program that is both unique and handsome and an interesting meeting is anticipated.

---

THE National Confederation of State Medical Examining and Licensing Boards will hold its next meeting at Columbus, June 5, 1899, beginning at 10 o'clock a. m. It is composed of members of the various state medical licensing and examining boards and its officers are as follows: President, William Warren Potter, Buffalo, N. Y.; vice-presidents, E. L. B. Godfrey, Camden, N. J.; William Bailey, Louisville, Ky.; secretary-treasurer, A. Walter Suiter, Herkimer, N. Y.; executive council, chairman, William S. Foster, Pittsburg, Pa.; Joseph M. Mathews, Louisville, Ky.; Hugh M. Taylor, Richmond, Va.; W. H. Saunders, Montgomery, Ala.; Charles K. Cole, Helena, Mont.; committee on minimum standard of requirements, chairman, N. R. Coleman, Columbus, O.; Gardiner Swarts, Providence, R. I.; T. J. Happel, Trenton, Tenn.; Augustus Korndoerfer, Philadelphia, Pa; W. R. Tipton, Las Vegas, N. M. The sessions will be held in the Senate Chamber, State Capitol. Governor Asa Bushnell will deliver the address of welcome on behalf of the state, and Dr. C. A. L. Reed, Cincinnati, on behalf of the physicians of Ohio. President James Canfield, of the Ohio State University, will deliver an address before the meeting, entitled, An educational foundation for a physician's special training. The committee on minimum standard of requirements, will make its report. The work of this committee constitutes the most important object of the confederation, which is to elevate and make uniform the standard of requirements for admission to medical colleges, to harmonise, as far as possible and make parallel their courses of study and to discuss the best methods of conducting examinations for license to practise, in all the various states of the Union. Inasmuch as the membership of the confederation is made up of physicians who have positions on the official boards governing these matters in the several states, it can be seen that its deliberations will have considerable influence in medical legislation and the enforcement of such laws.

---

THE Alumni Association of the Medical Department of Niagara University held a meeting May 3, 1899, at the Buffalo Library building. The following officers were elected for the ensuing year: President, L. J. McAdam; first vice-president, J. P. F. Burke; second vice-president, M. A. Sullivan; secretary and treasurer, W. H. Norrish; executive committee, J. J. Finerty, L. G. Hanley, E. A. Forsyth.

The following papers were read and discussed: Uterine fibroids, C. E. Congdon; Neurasthenia from the standpoint of a general practitioner, C. W. Coulter, of Oil City, Pa.; Osteomyelitis, E. M. Dooley. The annual banquet was held in the evening at the New Gruener Hotel.

